# Comparison of in-person vs. telebehavioral health outcomes from rural populations across America

**DOI:** 10.1186/s12888-022-04421-0

**Published:** 2022-12-10

**Authors:** Carly McCord, Fred Ullrich, Kimberly A. S. Merchant, Divya Bhagianadh, Knute D. Carter, EveLynn Nelson, James P. Marcin, Kari Beth Law, Jonathan Neufeld, Annaleis Giovanetti, Marcia M. Ward

**Affiliations:** 1 Department of Psychiatry and Behavioral Sciences, Texas A&M University, College Station, TX USA; 2 Department of Educational Psychology, Texas A&M University, College Station, TX USA; 3 Department of Health Management and Policy, University of Iowa, Iowa City, IA USA; 4 School of Social Work, Rutgers University, New Brunswick, NJ USA; 5 Department of Biostatistics, University of Iowa, Iowa City, IA USA; 6 Department of Pediatrics, University of Kansas Medical Center, Kansas City, KS USA; 7 Department of Pediatrics, University of California, Davis School of Medicine, Sacramento, CA USA; 8 Department of Behavioral Medicine and Psychiatry, West Virginia University, Morgantown, WV USA; 9 Institute for Health Informatics, University of Minnesota, Minneapolis, MN USA; 10 Department of Psychology, University of Kansas, Kansas City, KS USA

**Keywords:** Telehealth, Behavioral health, Mental health, Rural, PHQ-9, GAD-7

## Abstract

**Background:**

This study investigates outcomes from two federal grant programs: the Evidence-Based Tele-Behavioral Health Network Program (EB THNP) funded from September 2018 to August 2021 and the Substance Abuse Treatment Telehealth Network Grant Program (SAT TNGP) funded from September 2017 to August 2020. As part of the health services implementation program, the aims of this study were to evaluate outcomes in patient symptoms of depression and anxiety across the programs’ 17 grantees and 95 associated sites, with each grantee having data from telehealth patients and from an in-person comparison group.

**Methods:**

The research design is a prospective multi-site observational study. Each grantee provided data on a nonrandomized convenience sample of telehealth patients and an in-person comparison group from sites with similar rural characteristics and during the same time period. Patient characteristics were collected at treatment initiation, and clinical outcome measures were collected at baseline and monthly. The validated clinical outcome measure instruments included the Patient Health Questionnaire-9 (PHQ-9) for depression symptoms and the Generalized Anxiety Disorder-7 (GAD-7) scale for anxiety-related symptoms. Linear mixed models, with grantee as the random effect, were used to determine the association of behavioral health delivery (telehealth versus in-person) on the one-month change in PHQ-9 and GAD-7 while adjusting for covariates.

**Results:**

Across a total of 1,514 patients, one-month change scores were improved indicating that PHQ-9 and GAD-7 scores decreased from baseline to the one-month follow-up at similar rates in both the in-person and telehealth groups. Reduction in scores averaged 2.8 for the telehealth treatment group and 2.9 for the in-person treatment group in the PHQ-9 subsample and 2.0 for the telehealth treatment group and 2.4 for the in-person treatment group in the GAD-7 subsample. There was no statistically significant association between the modality of care (telehealth treatment group versus in-person comparison group) and the one-month change scores for either PHQ-9 or GAD-7. Individuals with higher baseline scores demonstrated the greatest decrease in scores for both measures. Upon adjusting for baseline scores and grantee program, patient demographics were not found to be significantly associated with change in anxiety or depression symptoms.

**Conclusion:**

In our very large pragmatic study comparing behavioral health treatment delivered to a population of patients in rural, underserved communities, we found no clinical or statistical differences in improvements in depression or anxiety symptoms as measured by the PHQ-9 and GAD-7 between patients treated via telehealth or in-person.

## Background

Rural residents are particularly vulnerable to behavioral health disparities in accessibility and availability of treatment and in treatment outcomes [1, 2]. These disparities are multifactorial including distance and topographical challenges in getting to healthcare facilities, lower rates of insured patients, higher rates of poverty, and lower education and health literacy rates [3, 4]. Challenges may be multiplied for women and minority groups facing additional barriers related to childcare, actual and perceived stigma, and discrimination within the healthcare system [5]. The COVID-19 pandemic has further exacerbated the inequities that exist in social determinants of health seen in rural communities, which contribute to disparities in mental health outcomes [6].

Telehealth has been used to address some of these disparities, particularly related to access and distance to healthcare professionals [7]. Behavioral health care has been a leader in the utilization of telehealth applications given that evaluation and treatment typically translate easily between visit encounter modalities (telephone, videoconference, in-person) [8]. Randomized control trials (RCTs) and subsequent reviews demonstrate the efficacy of telebehavioral health and have failed to detect significant differences between in-person and telehealth interventions [9, 10]. However, many previous studies have limited generalizability and frequently lack diversity in patient populations with rural and minority patients often being underrepresented [11]. More pragmatic research is needed to understand effectiveness in real world settings and specifically among rural populations. Treatment as usual models can provide additional evidence for outcomes that reflect the heterogeneous reality of treatment with greater ecological validity [12, 13].

The current study helps address this gap in the literature. The Health Resources and Services Administration (HRSA) in the U.S. Department of Health and Human Services strategically funds programs that improve health outcomes and work to achieve health equity in rural and underserved communities. This study investigates outcomes from two grant programs: the Evidence-Based Tele-Behavioral Health Network Program (EB THNP) funded from September 2018 to August 2021 and the Substance Abuse Treatment Telehealth Network Grant Program (SAT TNGP) funded from September 2017 to August 2020. The two-fold purpose of these programs was to (1) increase access to behavioral health care services in rural and frontier communities, and (2) conduct evaluations of those efforts to establish an evidence base. Seventeen grantees implemented telebehavioral health services and/or substance abuse treatment in a manner consistent with the needs, resources, and capacity within their communities and health systems. Telehealth services were conducted using synchronous, audio-visual modalities. The interventions were not standardized and the sample represents an eclectic set of evidence-based/informed behavioral health interventions and service delivery models provided to rural residents across the United States.

With almost one-third of American adults reporting symptoms consistent with an anxiety or depression diagnosis (and prevalence highs during the pandemic were over 40%), these presenting concerns are common in the community setting, have evidence-based/informed treatments, and have a significant impact on morbidity, quality of life, and mortality [14]. While the pandemic has exacerbated underlying mental health issues for many Americans, barriers to receiving mental health care have existed for years. The aims of this study were to evaluate outcomes in depression and anxiety symptoms across all sites and compare outcomes between in-person and telehealth groups. It was hypothesized that there would be no clinically (5 points or greater) or statistically significant differences in outcomes between the in-person and telehealth groups.

## Methods

### Sample

The EB THNP funded 14 grantees and the SAT TNGP funded 3 grantees. The 17 telehealth network grantees provided behavioral health services to 95 clinic sites serving rural communities (as defined by the Office of Management and Budget (OMB)) in California, Indiana, Kansas, Kentucky, Maryland, Massachusetts, Minnesota, Missouri, Oregon, Pennsylvania, South Dakota, Texas, and West Virginia. The organizations are diverse in their workforce including but not limited to psychiatrists, psychologists, licensed professional counselors, and clinical social workers. Sites represent Affordable Care Organizations (ACOs), Community Health Centers (CHCs), and academic medical centers. Samples of participants were chosen by grantees to reflect the priorities identified in each respective funded project. All projects were similar in that they were providing both in-person and telehealth, behavioral health services and were serving rural and underserved populations. This resulted in a multi-state rural patient sample.

### Instruments

Concurrently, HRSA’s Office for the Advancement of Telehealth (OAT) funded the Rural Telehealth Research Center (RTRC) to serve as a data coordinating center for the programs. RTRC conducted a literature review to identify candidate data collection measures, reviewed and scored measures with experts and the grantees, honed the candidate measures to a final set, and operationalized the measures into 26 component data elements [15]. Data elements included patient characteristics (e.g., age, sex, race, primary insurance type), treatment group (telehealth versus in-person), clinician type (e.g., psychiatrist, psychologist, social worker), CPT/HCPCS codes, and clinical instruments as outcome measures. The clinical outcome measure instruments included the Patient Health Questionnaire-9 (PHQ-9) for depression symptoms [16] and the Generalized Anxiety Disorder-7 (GAD-7) scale for anxiety-related symptoms [17].

RTRC created a manual of operations including a dictionary of all data elements to define terms, indicate allowable values, and provide abstractor notes [15]. In addition to a training manual, an Excel-based tool, termed the Behavioral-Telehealth Evidence Collection Tool (B-TEC Tool), was created for data collection. Data Transfer and Use Agreements (DTUAs) were established between RTRC and each grantee. The research protocol was reviewed by the University of Iowa Institutional Review Board who approved it as Not Human Subject Research “because no protected health information was involved and data were deidentified prior to transmission to RTRC.” To facilitate both the signing of the DTUAs and Institutional Review Board approval, no protected health information was involved and data were de-identified prior to transmission to RTRC. RTRC performed data monitoring and management activities to verify data accuracy, completeness, consistency, and timeliness. OMB clearance was received in October 2019, and grantees provided data from then until July 23, 2021 [15]. All grantees from each grant program (SAT TNGP and EB THNP) enrolled patients in a rolling fashion. Each patient included within the sample was evaluated based upon the individual’s first three months of treatment. As such, the study duration for each patient (3 months) was the same; however, these did not all occur in a concurrent fashion.

### Procedure

A cross-grantee protocol was established which defined sample parameters and timeframes. With the nonrandomized prospective design, the telehealth sample was to include data from all patients who began telehealth treatment as part of either grant program during the data collection period. A comparison sample was to include data from a similarly matched group of patients who began in-person treatment. Grantees were asked to identify patients for the in-person comparison sample who had similar demographics, primary complaint or diagnosis, and who received comparable treatment (e.g., therapeutic approach and clinician type). Data collection for any patient was to extend for up to three months after treatment initiation and include data on all encounters during that period. Patient characteristics were to be collected at treatment initiation, and clinical outcome measures were to be collected at baseline and monthly on patients where appropriate. The RTRC specified inclusion and exclusion criteria for assessment administration stating that the PHQ-9 was to be administered to patients presenting with depressive symptoms or depression diagnoses, and the GAD-7 was to be administered to patients presenting with anxiety complaints or anxiety diagnoses. The clinical outcome measures were not to be utilized for patients where either measure was clinically uninformative or irrelevant (i.e. for patients not presenting with anxiety or depression).

### Data Analysis

The analyses included only patients who had valid scores for either clinical outcome instrument (PHQ-9: 0–27, GAD-7: 0–21) and who were administered a baseline assessment within two weeks before or after initiating treatment, and follow-up assessments within 16 weeks of initiating treatment. Baseline characteristics of patients in the telehealth treatment group and the in-person treatment group were compared using chi-squared tests. Regression models (linear for continuous variables, logistic for dichotomous) were used to test for differences between the telehealth treatment and the in-person treatment groups. The primary outcome was the change score from baseline to assessments at one month after treatment initiation. A one-month score change was used as the primary outcome to minimize sample attrition from longer follow-up periods which were investigated as sensitivity analyses. Linear mixed models, with grantee as the random effect, were used to determine the association of behavioral health delivery (telehealth versus in-person) on the one-month change in PHQ-9 and GAD-7 while adjusting for fixed effect covariates (age group, sex, race, ethnicity, and insurance status). Sensitivity analyses examined the change scores using categorical values. For the categorical sensitivity analyses, the PHQ-9 categories included no depression (0), minimal depression [1–4], mild depression [5–9], moderate depression [10–14], moderately severe depression [15–19], and severe depression [20–27]. The GAD-7 categories included minimal anxiety (0–4), mild anxiety [5–9], moderate anxiety [10–14], and severe anxiety [15–21]. Additional sensitivity analyses computed the regression models using change scores from baseline to months two and three. In another sensitivity analysis, we controlled for the number of encounters in a month (mean number of encounters 2.33 in the PHQ-9 subsample and 2.35 in the GAD-7 subsample) in the models.

## Results

There were 770 patients in the telehealth treatment group and 752 patients in the in-person treatment group in the final PHQ-9 subsample. For the GAD-7 subsample, there were 638 patients in the telehealth treatment group and 652 patients in the in-person treatment group. Altogether, there were 1,574 unique patients in the study. Of these 1,238 (79%) patients were present in both PHQ-9 and GAD-7 subsamples. This overlap group constituted 81% and 96% of the PHQ-9 and GAD-7 subsample respectively. As shown in Table 1, the patients in the telehealth treatment group differed significantly from the patients in the in-person treatment group within both the PHQ-9 and GAD-7 subsamples for age, sex, race, ethnicity, and insurer. In both subsamples, the patients in the telehealth treatment group were more likely under 18 or over 65 years old, female, white, non-Hispanic/non-Latinx, and insured by Medicaid or dual Medicare/Medicaid as compared to the in-person treatment group.

**Table 1:** Baseline characteristics of patients in the PHQ-9 and GAD-7 sub-samples

Sub-samples	PHQ-9 Sub-sample					GAD-7 Sub-sample				
	Telehealth treatment group *N* = 770		In-person control group *N* = 752			Telehealth treatment group *N* = 638		In-person control group *N* = 652		
	N	%	N	%	*p*-value	N	%	N	%	*p*-value
Age										
0–18	148	19.2	73	9.7	0.001	135	21.2	64	9.8	0.001
19–34	226	29.4	256	34.0		178	27.9	226	34.7	
35–64	309	40.1	351	46.7		250	39.2	301	46.2	
65 +	87	11.3	72	9.6		75	11.7	61	9.4	
Sex										
Female	546	70.9	497	66.1	0.005	451	70.7	425	65.2	0.01
Male	224	29.1	248	33.0		187	29.3	221	33.9	
Other/prefer not to say/unknown	0	0.0	7	0.9		0	0.0	6	0.9	
Race										
Asian/American Indian/Alaska Native/Native Hawaiian/Other Pacific Islander	13	1.7	14	1.9	0.001	12	1.9	12	1.8	0.001
Black/African American	24	3.1	21	2.8		22	3.5	17	2.6	
White	677	87.9	593	78.9		560	87.8	502	77.0	
Multi-racial	10	1.3	26	3.5		11	1.7	26	4.0	
Unknown	46	6.0	98	13.0		33	5.2	95	14.6	
Ethnicity										
Hispanic/Latinx	50	6.5	38	5.1	0.001	40	6.3	37	5.7	0.001
Not Hispanic/not Latinx	676	87.6	619	82.3		560	87.8	520	79.8	
Unknown	44	5.7	95	12.6		38	6.0	95	14.6	
Insurance Status										
Medicaid	243	31.6	209	27.8	0.001	209	32.8	188	28.8	0.001
Medicare	97	12.6	102	13.6		79	12.4	92	14.1	
Dual Medicare/Medicaid	32	4.2	11	1.5		31	4.9	9	1.4	
Private Insurance	318	41.3	295	39.2		244	38.2	233	35.7	
Self-pay/uninsured	50	6.5	63	8.4		43	6.7	58	8.9	
Other	19	2.5	12	1.6		19	3.0	11	1.7	
Unknown	11	1.4	60	8.0		13	2.0	61	9.4	

*Notes.* Percentages may not sum to 100% due to rounding

*P*-values obtained from chi-square tests comparing telehealth treatment group and in-person control group within each sub-sample

One-month change scores were improved indicating that PHQ-9 and GAD-7 scores decreased from baseline to the one-month follow-up. These averaged a decrease of 2.8 for the telehealth treatment group and 2.9 for the in-person treatment group in the PHQ-9 subsample and a decrease of 2.0 for the telehealth treatment group and 2.4 for the in-person treatment group in the GAD-7 subsample.

**Fig. 1:**
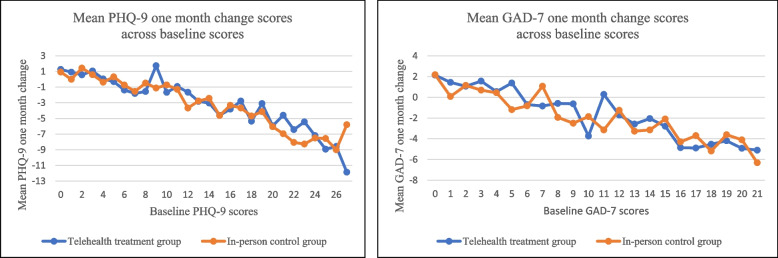
Mean one month change scores across baseline scores for telehealth treatment and in-person comparisongroups. Note. PHQ-9 has a maximum score of 27 and is shown in the left panel. GAD-7 has a maximum score of 21 and is shown in the right panel

As shown in Fig. 1, change scores were highly related to baseline scores, with patients having higher baseline scores on each measure showing greater improvement (decreases in scores) at one month. Because of this strong pattern, baseline scores were included in linear regression models in addition to the patient demographics selected *a priori*.

As shown in Table 2, one-month change scores were significantly related to baseline scores both within the PHQ-9 subsample model and within the GAD-7 subsample model. After adjusting for baseline scores and grantee, none of the patient demographics were significant in either linear mixed model. Importantly, there were no significant associations between the modality of care (telehealth treatment group versus in-person treatment group) and the one-month change scores for either PHQ-9 or GAD-7.

**Table 2:** Mixed linear regression models with random effects at grantee level predicting change at one month in PHQ-9 score (shown on the left) and GAD-7 score (shown on the right)

**Variable**	PHQ-9 Score Change^a^			GAD-7 Score Change^a^		
	**Coefficient**	*P* **-value**	**95% CI**	**Coefficient**	*P* **-value**	**95% CI**
Telehealth group^b^	0.05		-0.53, 0.62	0.19		-0.35, 0.74
Baseline score	-0.38	0.001	-0.42, -0.34	-0.35	0.001	-0.40, -0.31
*Age group (0–18 as reference)*						
Age group 19–34	-0.58		-1.54, 0.38	-0.23		-1.08, 0.62
Age group 35–64	-0.77		-1.70, 0.16	-0.31		-1.13, 0.50
Age group 65+	-1.48	0.05	-2.70, -0.27	-0.95		-2.03, 0.13
*Sex (Male as reference)*						
Female	0.42		-0.15, 0.99	0.80	0.01	0.23, 1.36
*Race (White as reference)*						
Non-Whites	0.30		-0.74, 1.35	0.35		-0.65, 1.35
Unknown race	-0.55		-1.59, 0.49	-0.34		-1.31, 0.63
*Ethnicity (Hispanic as reference)*						
Non-Hispanic	-0.18		-1.46, 1.10	-0.02		-1.23, 1.18
Unknown ethnicity	-0.39		-1.93, 1.15	-0.24		-1.65, 1.17
*Insurance status (Private ins. as reference)*						
Public insurance	0.29		-0.36, 0.95	0.37		-0.22, 0.96
Self-pay/unknown /other insurance	0.19		-0.76, 1.15	0.01		-0.82, 0.80
**N**	**1,514**			**1,284**		

^a^Both models adjust for age, gender, race, ethnicity, insurance type, and baseline score. Additionally, random effects are specified at the grantee level. None of the covariates are significantly related to the outcome variable (last follow-up change scores) other than baseline scores in both models and self-pay/unknown/other insurance in the subsample model

^b^Telehealth treatment group is a binary variable coded as 1 for patients in the telehealth treatment group and as 0 for patients in the in-person control group

For the sensitivity analyses, baseline scores were grouped to make a categorical variable and found the same pattern of results. Additional sensitivity analyses where the change in scores were calculated from baseline to two months and from baseline to three months and where we controlled for the number of encounters in a month demonstrated consistent results. That is, the modalities of care (telehealth versus in-person) were equally associated with reductions in PHQ-9 and GAD-7.

## Discussion

In our very large pragmatic study comparing behavioral health treatment delivered to a population of patients in rural, underserved communities, we found no clinical or statistical differences in improvements in depression or anxiety symptoms as measured by the PHQ-9 and GAD-7 between treatments delivered via telehealth or in-person. Results were as anticipated with patients in both treatment groups improving in symptoms over time and at similar rates. Additionally, as anticipated in similar community samples, individuals with higher baseline scores on both measures demonstrated the greatest decrease in scores. After adjusting for baseline scores and grantee, patient demographics were not found to be significantly associated with change in anxiety or depression symptoms.

Telehealth utilization began over 60 years ago with many historical publications representing specific applications of telepractice with outcomes related to satisfaction and feasibility [18]. As the impetus for evidence-based practice strengthened, the last 20 years of research has maintained a strong focus on treatment efficacy and randomized control trials as the gold standard methodology [19]. Today, critiques about generalizability and lack of diverse samples have driven the field to build evidence of effectiveness of more widespread applications of telehealth using symptom self-report measures as outcome variables [13, 20]. This study fills an important gap investigating anxiety and depression outcomes in telehealth and in-person samples across 17 partner organizations, at 95 clinical sites, in 13 states and over 1,000 participants with significant rural applicability.

This study found that an average of a three-point reduction in anxiety and depressive symptoms is achieved from the initial score to one-month following intervention regardless of whether the modality for treatment was in-person or via telehealth. This is consistent with meta-analyses that examined direct comparison RCTs of telehealth and in-person psychotherapy and found similar efficacy between delivery modalities for both anxiety [21] and depression [9].

### Limitations and future directions

The primary limitation of this nonrandomized study is the lack of a systematically matched control population in the types and timing of treatments across the sites. However, while the in-person cohort had differences from the telehealth cohort that could have led to limits in internal validity, we conducted our analyses adjusting for measured and known confounders. This pragmatic approach helps maximize external validity. Further, our sensitivity analyses considering different time frames of treatment consistently demonstrated that the results of care were independent of the modality of care. Because treatment was not standardized, a clear picture of any specific interventions and fidelity to evidence-based/informed approaches that improve anxiety and depression symptoms cannot be obtained. The focus of the study was to investigate the influence of modality on outcomes, and no statistically significant difference wasdetected.

The study assessed the important one-month time point related to symptoms of anxiety and depression. Future studies are needed to understand similarities/differences between telehealth and in-person care on longer term engagement in interventions, “dose” of interventions, and long-term outcomes on behavioral symptoms and functioning. Future trials may also explore if/which patient populations especially benefit from telehealth or may have challenges with telehealth, as well as if other factors (e.g., location of telehealth, linkages to primary care, other) enhance outcomes.

The impact of significant differences between the in-person and telehealth groups at baseline is unclear. Future mixed methods trials may explore these factors further and better understand patient uptake of telehealth services. The future directions of telehealth research should explore which variables explain or predict change better than modality and narrowing for whom particular treatments are most effective. For example, future research may explore system, provider, patient, and process variables in addition to outcome variables. Mixed methods studies and the use of dissemination and implementation science models are important next steps to give greater context to patient, organization, provider, and community influences on observed outcomes. Future research should continue to strive to include underrepresented groups at risk for behavioral health disparities. While this study included rural-residing individuals who are often underrepresented in research, the sample was largely white.

The grantees were awarded grants related to pre-pandemic proposals and quickly had to shift to meet the overwhelming needs and new realities of the pandemic. This impacted shifts from clinic-based to home-based telebehavioral health delivery due to social distancing needs and loosening of telehealth policy requirements during the public health emergency. The overall findings suggest the benefit of both in-person and telehealth services even with updates in how this care may have been delivered. Future studies may continue to assess the equivalence of the two groups—in-person and telehealth—in non-pandemic conditions.

Depression and anxiety are most commonly treated in outpatient settings like those in this study with an ever-growing push towards treatment in primary care settings [22, 23]. Studies indicate that anxiety and depression rates are especially high (between 39% and 44%) in rural primary care settings [24–26]. Despite the high need for mental health care in these settings, primary care physicians have reported difficulty referring patients to mental health providers in part due to shortages [27]. More than 60% of rural Americans live in a mental Health Professional Shortage Area, and more than 90% of psychologists and psychiatrists and 80% of social workers practice exclusively in metropolitan areas [28]. This highlights an important area for future research examining the utility of telehealth in addressing access gaps and unmet needs in these settings.

### Policy implications

Knowing that telebehavioral health is effective on a large scale for the debilitating and costly conditions of anxiety and depression, several important policy implications can be gleaned from these findings including support for parity in payment between telehealth and in-person care. Coverage parity refers to equivalent insurance coverage for patients, whereas payment parity encompasses provider reimbursement. As of mid-April 2022, 50 states and Washington, D.C. provide reimbursement for some form of live video in Medicaid fee-for-service. Forty-three states and D.C. have a private payer law that addresses telehealth reimbursement. Not all of these laws require reimbursement or payment parity. Twenty-one states have explicit payment parity. There continue to be uncertainties around the Centers for Medicare and Medicaid Services (CMS) and the telehealth policy landscape post public health emergency. More specific outcome data from pragmatic trials such as this can help inform policy decisions, such as rules around mental health reimbursement and in-person visits after the official end of the public health emergency. Of particular relevance to this study, 31 states have enacted PSYPACT currently, with additional states pending legislation [29]. PSYPACT is an interstate compact designed to facilitate the practice of telepsychology, expanding potential telebehavioral workforce to meet growing behavioral health needs across the lifespan, particularly in rural areas.

As payment parity continues and restrictions on urban use of telehealth are removed, policy must find ways to incentivize providers to continue to work in rural areas where the logistical and ethical challenges to providing care are significant. Other factors also highlight the continued need to provide focused attention and funding on serving rural and frontier areas [30]. Human nature and business imperatives for profit may unintentionally steer telehealth service delivery through the path of least resistance and inadvertently increase disparities in rural areas.

Workforce development remains a critical issue nationally and is worst in rural areas [31]. Low behavioral health provider supply is even more acute in non-core counties: 80% lacked a psychiatrist; 61% lacked a psychologist; and 91% lacked a psychiatric nurse practitioner [32]. Moreover, rural areas have been disproportionately impacted by the public health emergency, with significant negative impacts on unemployment, overall life satisfaction, mental health, and economic outlook [33], making telehealth and other access options even more pressing.

## Conclusion

The investment by organizations and governments in telehealth in rural areas has been significant and has had a positive impact on countless communities, families, and individuals. The disease burden of mental illness is the highest among all diseases [34] and improving access to care through telehealth is an important component of the solution to a complex problem. The partner organizations increasing access to care in this study represent diverse applications of telehealth. Some organizations have clinicians in rural sites providing care to other rural areas and others have urban hubs providing care to rural areas. The organizations are diverse in their workforce including but not limited to psychiatrists, psychologists, licensed professional counselors, and clinical social workers. They represent Affordable Care Organizations, Community Health Centers, and academic medical centers. This study suggests that the telebehavioral health care delivered to rural areas is as effective as in-person care for anxiety and depression symptoms, an important reassurance with the continued expansion of telehealth beyond the pandemic surge in telehealth adoption.

## Data Availability

Data for this study are maintained by the Rural Telehealth Research Center (RTRC). All data requests should be referred by email to rtrc-inquiry@uiowa.edu and the RTRC Executive Committee will review and respond to all requests. Data for this project were obtained under restrictive DTUAs between the University of Iowa researchers and each service network which, in turn, received their data under DTUAs with their participating clinic sites. Those DTUAs stipulate that the data, other than statistical summary information such as in this paper, cannot be shared publicly as they contain potentially identifying and sensitive patient information.
